# Influence of Isoflurane on Immediate-Early Gene Expression

**DOI:** 10.3389/fnbeh.2015.00363

**Published:** 2016-01-12

**Authors:** Kristopher M. Bunting, Rebecca I. Nalloor, Almira Vazdarjanova

**Affiliations:** ^1^Department of Pharmacology and Toxicology, Medical College of Georgia, Augusta University, AugustaGA, USA; ^2^Vazdarjanova Lab, Research Department, Charlie Norwood VA Medical Center, AugustaGA, USA

**Keywords:** isoflurane, Arc, Zif268, immediate early gene, catFISH

## Abstract

**Background:** Anterograde amnesia is a hallmark effect of volatile anesthetics. Isoflurane is known to affect both the translation and transcription of plasticity-associated genes required for normal memory formation in many brain regions. What is not known is whether isoflurane anesthesia prevents the initiation of transcription or whether it halts transcription already in progress. We tested the hypothesis that general anesthesia with isoflurane prevents learning-induced initiation of transcription of several memory-associated immediate-early genes (IEGs) correlated with amnesia; we also assessed whether it stops transcription initiated prior to anesthetic administration.

**Methods:** Using a Tone Fear Conditioning paradigm, rats were trained to associate a tone with foot-shock. Animals received either no anesthesia, anesthesia immediately after training, or anesthesia before, during, and after training. Animals were either sacrificed after training or tested 24 h later for long-term memory. Using Cellular Compartment Analysis of Temporal Activity by Fluorescence *in situ* Hybridization (catFISH), we examined the percentage of neurons expressing the IEGs *Arc/Arg3.1* and *Zif268/Egr1/Ngfi-A/Krox-24* in the dorsal hippocampus, primary somatosensory cortex, and primary auditory cortex.

**Results:** On a cellular level, isoflurane administered at high doses (general anesthesia) prevented initiation of transcription, but did not stop transcription of *Arc* and *Zif268* mRNA initiated prior to anesthesia. On a behavioral level, the same level of isoflurane anesthesia produced anterograde amnesia for fear conditioning when administered before and during training, but did not produce retrograde amnesia when administered immediately after training.

**Conclusion:** General anesthesia with isoflurane prevents initiation of learning-related transcription but does not stop ongoing transcription of two plasticity-related IEGs, *Arc* and *Zif268*, a pattern of disruption that parallels the effects of isoflurane on memory formation. Combined with published research on the effects of volatile anesthetics on memory in behaving animals, our data suggests that different levels of anesthesia affect memory via different mechanisms: general anesthesia prevents elevation of mRNA levels of *Arc* and *Zif268* which are necessary for normal memory formation, while anesthesia at lower doses affects the strength of memory by affecting levels of plasticity-related proteins.

## Introduction

Inhaled anesthetics have been used for general anesthesia for more than 160 years (Long, 1849); however, the mechanisms of action of anesthetic agents have remained largely unknown until the past several decades. Of the two basic endpoints of general anesthesia, immobility to noxious stimuli and a reversible state of amnesia (Eger et al., 1997), anesthetic-induced amnesia is of particular importance. Patients anesthetized for surgery sometimes become aware during surgery and have horrific memories of their experience, despite the appearance of being under adequate anesthesia (Kent et al., 2013; Rule and Reddy, 2014; Mashour and Avidan, 2015). A better understanding of the molecular mechanisms of anesthetics on memory-forming processes can lead not only to academic enlightenment, but also to improved patient outcomes. To better understand these molecular mechanism, we examined the effect of general anesthesia with isoflurane on behavioral training in rats and the corresponding transcription of plasticity-related immediate-early genes (IEGs; Alkire et al., 2007; Alkire and Guzowski, 2008). Synaptic plasticity is the physical basis of memory, and the expression of certain plasticity-related IEGs, including *Arc* and *Zif268*, is required to form long-term memory (Dragunow, 1996; Bozon et al., 2002, 2003; Davis et al., 2003; Plath et al., 2006; Bramham et al., 2008, 2010; Nakayama et al., 2015). In a neurobiological context, learning is the formation of long-term memory and amnesia is the loss of previously acquired memory (retrograde amnesia) or the failure to form new long-term memories (anterograde amnesia). General anesthesia with isoflurane produces anterograde amnesia, but it is not clear at which stage of memory formation this occurs: acquisition of sensory data, processing of sensory input into short-term memory, or consolidation of long-term memory (Izquierdo et al., 1999). In other words, anterograde amnesia could be the result of failing to consolidate long-term memory or the failure to form any memory at all.

Isoflurane is known to suppress activity in neurons of the sensory thalamus in a dose dependent manner, but does not completely abolish neuronal activity (Vahle-Hinz et al., 2007). It is possible that amnesia occurs as a result of suppression of sensory input to the cerebral cortex and to the memory system in the medial temporal lobe, including the amygdala, hippocampus, and associated entorhinal and perirhinal cortex. However, simply suppressing neural activity does not necessarily reduce the number of cells engaged in activity-dependent plasticity-related IEG transcription. Recent research demonstrates that neuronal activity and IEG expression are not always coupled during learning (Guzowski et al., 2006; Carpenter-Hyland et al., 2010). Because not all neuronal firing induces activity-dependent IEG transcription, simply reducing neural activity does not necessarily reduce the size of neuronal ensembles engaged in this transcription. It is possible, however unlikely, that sensory input could result in neuronal activity that may induce synaptic plasticity and possibly lead to memory formation during the anesthetized state.

Knowing that general anesthesia with isoflurane induces anterograde amnesia, we wanted to delve deeper into the effects of isoflurane on biochemical markers of learning and memory. More importantly, we wanted to examine these effects in a temporally specific manner that would allow us to examine the formation of a specific memory, controlling for effects of sensory input and neuronal activity related to the induction of anesthesia. We developed a testable hypothesis that examines isoflurane’s effects downstream of its effect on neuronal firing, the most likely point at which anesthesia effects activity-dependent IEG transcription. We directly tested the hypothesis that general anesthesia with isoflurane will suppress learning-initiated transcription of the plasticity-related IEGs *Arc* and *Zif268* in brain regions involved in auditory fear conditioning: dorsal hippocampus, primary auditory cortex and primary somatosensory cortex (Romanski and LeDoux, 1992; Quirk et al., 1997; Bushnell et al., 1999; Maren and Holt, 2000; Anagnostaras et al., 2001; Kim and Jung, 2006). We further differentiated between the effect of isoflurane on initiation of transcription and interruption of transcription that is already underway. This dissociation is possible using the powerful molecular imaging and analysis technique known as Cellular Compartment Analysis of Temporal Activity by Fluorescence *in situ* Hybridization (catFISH; Guzowski et al., 2001) in conjunction with isoflurane anesthesia induced either before or immediately after Pavlovian fear conditioning.

The catFISH method allows us to examine gene expression with cellular resolution and temporal specificity when used with genes which have a short duration of transcription: *Arc* and *Zif268* (Guzowski et al., 1999; Vazdarjanova et al., 2002b). Transcription of *Arc* and *Zif268* mRNA follows a distinctive activity-induced time course following plasticity-inducing events, such as learning or maximal electroconvulsive shock (MECS), allowing for high temporal resolution when visualized using Fluorescence *in situ* Hybridization (FISH) with full-length antisense RNA probes. Intranuclear foci of transcription for *Arc* and *Zif268* mRNA are first detectable 2 min after transcription is initiated; these foci appear first as tiny specks that grow into easily identifiable, intensely stained dots by 5 min. After 10 min, the intranuclear RNA no longer appears as foci; it diffuses into the nucleoplasm and at 15 min appears as perinuclear staining along the nuclear membrane as it migrates into the cytoplasm. By 30 min, *Arc* and *Zif268* mRNA appear as diffuse cytoplasmic staining which fills the soma. After 30 min, *Zif268* remains in the soma while *Arc* is transported into dendrites for targeted, synapse-specific local translation (Steward et al., 1998; Guzowski et al., 1999; Vazdarjanova et al., 2002a,b). Therefore, by identifying individual cells with clearly visible foci of transcription 6 min after fear conditioning, we can accurately evaluate the size of IEG-expressing neuronal ensembles activated by the training.

## Materials and Methods

### Animals

After obtaining approval from the Institutional Animal Care and Use Committee (Augusta University, Augusta, GA, USA), 25 male Sprague-Dawley rats (2 months old, 250–300 g) were obtained from Charles River Laboratories (Wilmington, MA, USA) and housed in a temperature controlled colony room with food and water freely available. The animals were maintained on a 12 h reverse light cycle (lights on at 7 PM, lights off at 7 AM); testing was performed during the dark (active) phase.

### Behavioral Procedures

**FIGURE 1:**
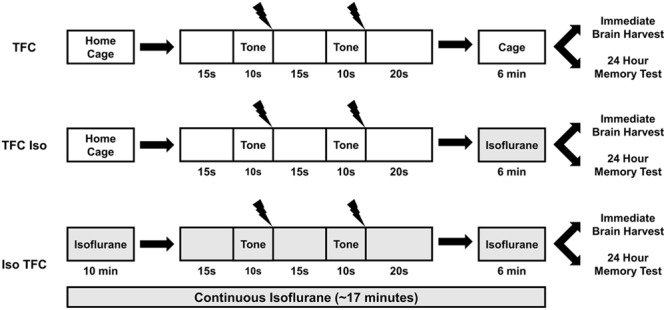
**Tone fear conditioning (TFC) and isoflurane administration.** This illustration depicts isoflurane administration (gray shaded areas) relative to TFC for each behavioral group. The TFC group was not anesthetized, the TFC-Iso group was anesthetized after TFC, and the Iso-TFC group was anesthetized before, during, and after TFC. Please see text for detailed experimental procedures.

The tone fear conditioning (TFC) training apparatus consisted of a 10 × 50 cm acrylic box with two opaque walls and transparent lid. The other two walls and floor were lined with two metal plates separated at the floor by a 1 cm gap. The animals were habituated to the apparatus 3 min per day for 5 days prior to training. On the training day, animals were placed into the apparatus for a total of 70 s and presented with two tones (10 s, 5 kHz, 80 dB) at 15 and 40 s, each co-terminating with a foot-shock (1 s, 0.7 mA AC). Foot-shock was delivered using a custom-made constant-current generator designed to deliver the full current across a single circuit, not a grid floor as used in commercially available fear conditioning units. At the current used, the shock is intense and unpleasant but not painful. **Figure 1** illustrates the behavioral parameters of each treatment group. Animals in the TFC group (*n* = 8) did not receive anesthesia, animals in the TFC-Iso group (*n* = 7) were anesthetized for 6 min immediately after training, and animals in the Iso-TFC group (*n* = 8) were anesthetized continuously from 10 min prior to training until 6 min after training. Freezing, defined as immobility except for respiration, was scored in non-anesthetized animals during training.

Three animals from each treatment group were sacrificed by decapitation 6 min after TFC training, while two caged controls were taken from their home cages and immediately sacrificed. The remaining rats (4–5 per group) were tested 24 h after training for long-term memory for fear conditioning. Fear conditioning to context and tone were tested separately 5 h apart. To assess contextual fear conditioning, animals were placed in the training apparatus for 5 min and freezing was scored. To assess conditioned fear to the tone alone, animals were placed in a novel context, a 46 × 37 cm box, for 2 min and then subjected to six 20 s presentations of the conditioned tone (5 kHz, 80 dB) played 10 s apart; animals were removed 2 min after the last tone. Freezing was scored prior to and during the tone presentation. The order of testing for fear conditioning to tone and fear conditioning to context was randomly assigned in a counterbalanced design. Freezing was scored by an observer blinded to each animal’s group assignment.

### Anesthetic Procedures

Anesthesia was induced by exposing animals to a vessel containing gauze saturated with isoflurane for 10–15 s, until loss of righting reflex was observed, and then maintained with 2% isoflurane delivered by a vaporizer with oxygen at a flow rate of 1.5–2 l/min via nose cone. The desired state of general anesthesia was defined as: loss of righting reflex, areflexia to a toe-pinch stimulus, and lack of purposeful movement except for spontaneous breathing. Animals were continuously observed while anesthetized and tested for toe-pinch response at regular intervals. Anesthetic was delivered at the minimum level required to maintain these criteria.

This induction method was designed to meet two criteria: first, to prevent the unwanted stimulation of learning pathways in the brain caused by exploration in a standard induction chamber; and second, to emulate the rapid induction of anesthesia practiced before euthanasia by decapitation. The study of time-sensitive IEG transcription necessitates tight control over the period several minutes before the subject’s brain is flash frozen.

### Fluorescence *in Situ* Hybridization

Animals were sacrificed, their brains quickly removed and immediately frozen. The frozen brains were sectioned using a cryostat microtome into 20 μm thick sections and mounted on microscope slides. The mounted tissue was fixed in 4% paraformaldehyde, permeabilized in a 1:1 solution of acetone and methanol, and then hybridized with digoxigenin-labeled *Arc* or *Zif268* full-length antisense RNA probes. These probes were synthesized using MAXIscript^®^ T3 and T7 (Ambion^®^, Austin, TX) and AmpliScribe^TM^ T7 (Epicentre Biotechnologies, Madison, WI, USA) RNA *in vitro* transcription kits. After peroxidase quenching and blocking, the hybridized tissue was incubated with peroxidase-conjugated anti-digoxigenin antibody (Roche, Indianapolis, IN, USA) and revealed using SuperGlo^TM^ Immunofluorescence Amplification Kits (Fluorescent Solutions, Augusta, GA, USA) or Tyramide Signal Amplification kits (Perkin Elmer, Waltham, MA, USA) with Cyanine 3 or Fluorescein. Nuclei were stained with SYTOX^®^ Green (Invitrogen, Carlsbad, CA, USA), DAPI, or 7-AAD.

### Imaging and Stereology

Stained slides were imaged using a Zeiss Axio Imager fluorescent microscope with ApoTome; multi-channel *z*-stack images were collected using a 20X/NA 0.8 objective.

Each 447 μm × 335 μm image stack consisted of 13–17 planes of 1.2 μm-thick optical sections. For each animal, at least two slides containing one section per slide were selected for each brain region; two image stacks were collected from each section. On average, four image stacks were analyzed per brain region for each animal; the total number of cells counted for each brain region by condition and probe are shown in Supplementary Table S1. Unbiased stereological cell counting was performed as follows: cells were classified as either neuron-like or glia-like based on their nuclear morphology; glia-like cells with small, intensely, and uniformly stained nuclei, were excluded from the analysis. Neuron-like cells in the regions of interest were counted using an optical dissector method, which minimizes sampling errors attributable to partial cells, as cell volumes do not influence sampling frequencies (West, 1999). Pairs of adjacent “lookup” and “sample” sections were stacked so that the first optical section in a stack was the first “lookup section,” and the second optical section was the first “sample section” as well as the “lookup section” for the next “sample section.” The dissector (the total number of “sample sections”) included the top 60% of a given stack, excluding the first “lookup section”. The dissector counting rule instructed that all neuron-like cells with leading edges present in each dissector should be included for further classification. This rule ensures that all neurons have equal probabilities of being included in the samples regardless of their size, because each of them is defined by their leading edge rather than their volume. It also minimizes type I classification errors where a positive partial cell is classified as negative because the portion of the cell containing the foci of transcription is missing. Neurons were classified as either positive or negative for intranuclear *Arc* or *Zif268* intranuclear foci visible on at least three optical planes. Results are expressed as the percentage of total neurons containing intranuclear foci of *Arc* or *Zif268* mRNA.

Anatomical regions were defined as follows (Paxinos and Watson, 2007): dorsal hippocampal CA1, Bregma -2.92 to -4.20 mm; primary auditory cortex A1, bregma -4.8 to -6.84 mm; primary somatosensory cortex S1, bregma -1.8 to -3.72 mm.

### Statistical Analysis

Data are reported as the mean ± the standard error of the mean (SEM). Because all data sets passed the test for normality, they were subjected to one-way analysis of variance (ANOVA) using the Tukey-Kramer *post hoc* test to compare differences between individual groups, or mixed-design Repeated-Measures ANOVA (RM-ANOVA) for the contextual fear conditioning data. The experiment wide alpha value was set at 0.05 and significant *post hoc* differences are reported when the mean difference between groups exceeded the critical difference calculated by the Tukey-Kramer test. Statistical analysis was performed using StatView software (SAS Institute, Cary, NC, USA).

## Results

### Behavior

**FIGURE 2:**
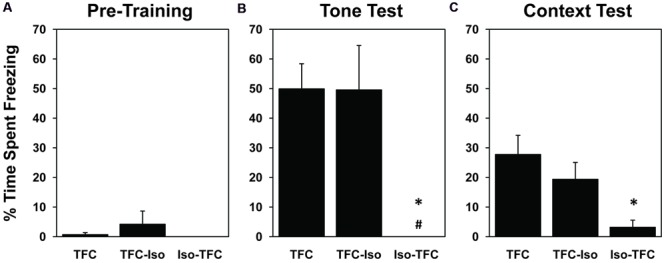
**Pre-training and post-training freezing.** During habituation to the foot-shock context prior to training **(A)**, all groups exhibited negligible freezing behavior. 24 h after TFC, animals were tested for freezing to tone in a novel context **(B)** and freezing in the foot-shock context without tone **(C)**. Both the TFC and TFC-Iso groups showed significantly higher levels of freezing to the tone compared with the Iso-TFC group **(B)**; while only the TFC group showed significantly higher levels of freezing to context compared with the Iso-TFC group **(C)**. These results show that animals anesthetized during training demonstrated anterograde amnesia for TFC, while animals anesthetized immediately after training showed no retrograde amnesia. ^∗^Statistical significance vs. TFC, #statistical significance vs. TFC-Iso.

Freezing behavior was used as a measure of memory for fear conditioning, as it indicates intense fear in rats (Blanchard et al., 1975, 1986). Pre-training freezing during the habituation period (**Figure 2A**) was low in all groups [mean = 1.85% ± 1.54, range = 0–21.67%, *F*(2,11) = 0.72, ns] showing that the training context by itself, before the tone-shock pairing, did not elicit fear. Pairing a tone with the foot-shocks resulted in conditioned fear to both the tone and the context in which the pairing occurred, as evidenced by elevated freezing in non-anesthetized animals (TFC group) during tone and context tests 24 h after training (50% ± 8.39 and 27.87% ± 6.33, respectively, **Figures 2B,C**). There was a significant effect of training on the RM-ANOVA for context [*F*(1,11) = 16.11, *p* = 0.002].

Significant differences were seen between groups in the post-training tone test [*F*(2,11) = 6.69, *p* = 0.01] and context test [*F*(2,11) = 5.02, *p* = 0.03]. Rats that received the same training as TFC rats while anesthetized (Iso-TFC group), showed significantly less freezing to tone and context (**Figures 2B,C**) when compared to the TFC group, indicating robust anterograde amnesia. There was no evidence of retrograde amnesia in rats anesthetized immediately after training (TFC-Iso group); these animals showed freezing comparable to the non-anesthetized TFC group in both tone and context testing (**Figures 2B,C**).

### Gene Expression

**FIGURE 3:**
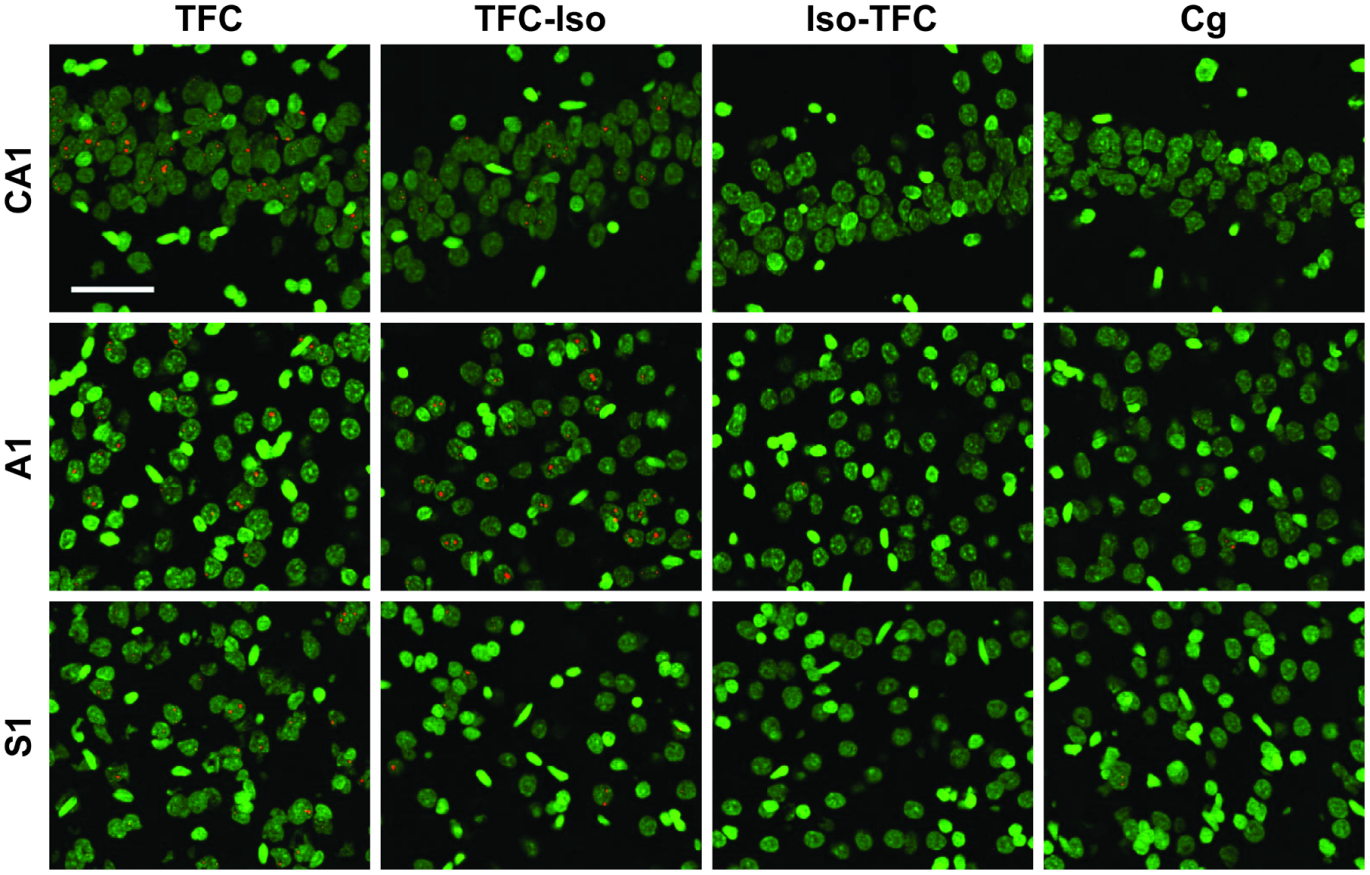
***Arc* catFISH.** Fluorescence photomicrographs of *in situ* hybridization with antisense riboprobes for *Arc* mRNA. *Arc* is shown in red, cell nuclei are stained with DAPI and psuedocolored green. Scale bar is 50 μm.

**FIGURE 4:**
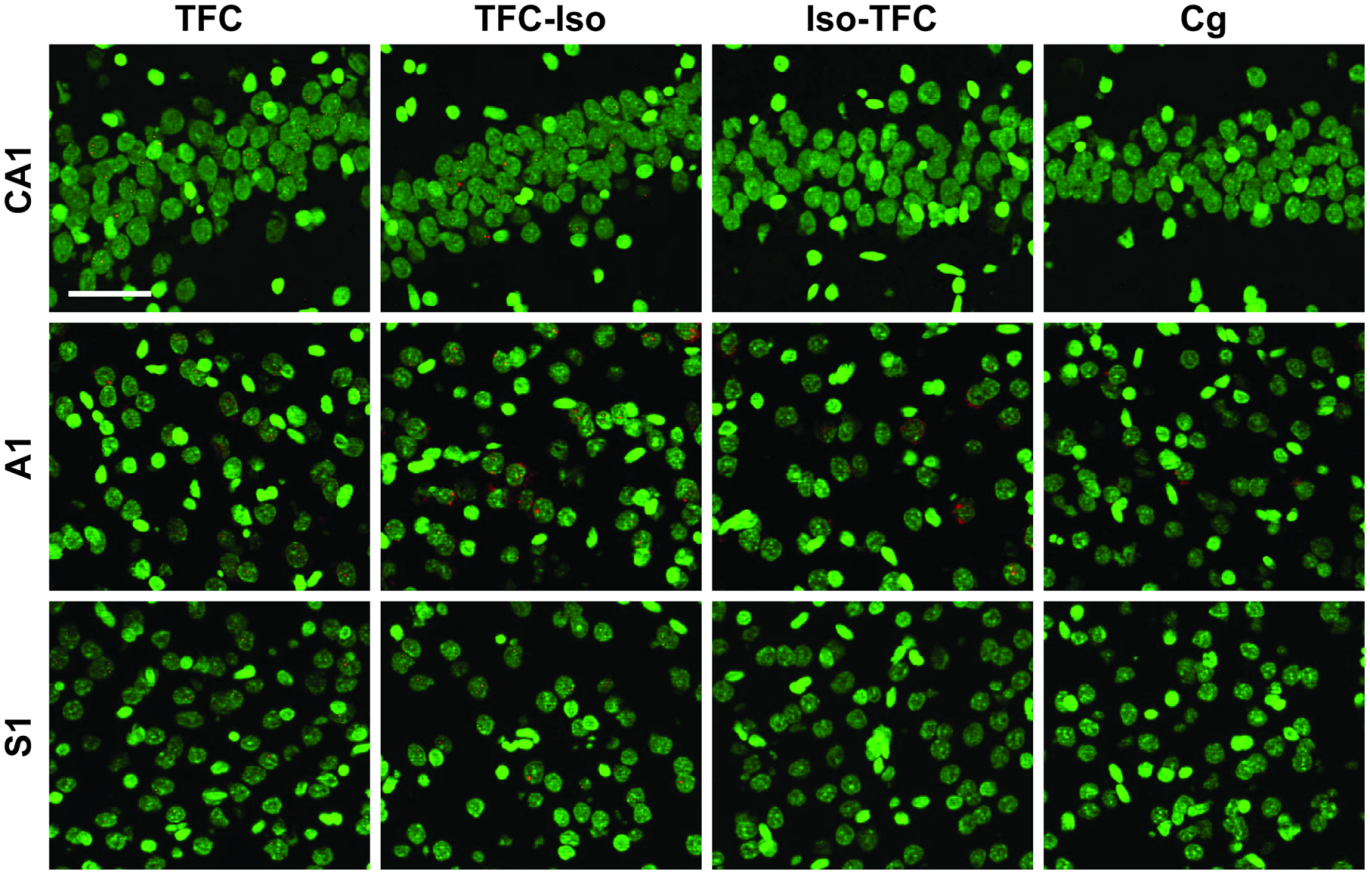
***Zif268* catFISH.** Fluorescence photomicrographs of *in situ* hybridization with antisense riboprobes for *Zif268* mRNA. *Zif268* is shown in red, cell nuclei are stained with DAPI and psuedocolored green. Scale bar is 50 μm.

**FIGURE 5:**
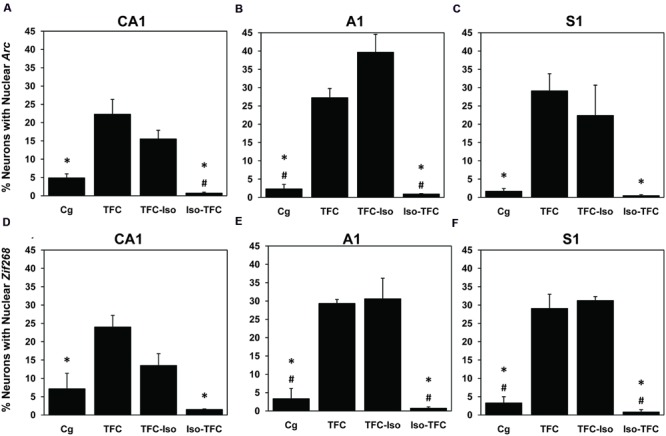
***Arc* and *Zif268* mRNA transcription.** Using the catFISH method, mRNA expression induced by TFC was measured and is reported as the percentage of neurons showing active transcription of the plasticity-related immediate-early genes (IEGs) *Arc* and *Zif268*. *Arc* transcription in Dorsal Hippocampal CA1 **(A)**, Primary Auditory Cortex A1 **(B)**, and primary somatosensory Cortex S1 **(C)** shows that the Iso-TFC group and caged controls both show significantly lower *Arc* expression than the TFC group. *Zif268* transcription in Dorsal Hippocampal CA1 **(D)**, Primary Auditory Cortex A1 **(E)**, and primary somatosensory Cortex S1 **(F)** shows the same pattern of significantly lower expression in the Iso-TFC and caged control groups compared with the TFC group. Compared with the TFC-Iso group, the Iso-TFC group showed significantly lower *Arc* expression in CA1 and A1, as well as significantly lower *Zif268* expression in A1 and S1. There was no significant difference between the TFC and TFC-Iso groups. The low levels of *Arc* and *Zif268* expression seen in the Iso-TFC group compared with both the TFC and TFC-Iso groups correlates with the lack of post-training freezing behavior observed in this group (**Figure 2**). General anesthesia administered during training prevented initiation of transcription of the learning-induced IEGs *Arc* and *Zif268* and caused anterograde amnesia, but general anesthesia administered immediately after training had no significant effect on learning or transcription of these IEGs initiated prior to anesthesia. General anesthesia with isoflurane does not directly interfere with ongoing IEG transcription. ^∗^Statistical significance vs. TFC, #statistical significance vs. TFC-Iso.

The percentage of neurons actively transcribing *Arc* and *Zif268* mRNA induced during TFC in three brain regions involved in tone and contextual fear conditioning is shown in **Table 1** and **Figure 5**. Representative images taken from analyzed stacks are shown for *Arc* in **Figure 3** and *Zif268* in **Figure 4**. For each gene, data for the CA1 region of the dorsal hippocampus was collected from an average of 491 neurons per rat (range: 351–822), 588 neurons per rat (range: 340–774) in primary auditory cortex (A1), and 636 neurons per rat (range: 356–998) in primary somatosensory cortex (S1).

**Table 1:** catFISH data – active *Arc* and *Zif268* transcription.

	*Arc*			*Zif268*		
Group	CA1	A1	S1	CA1	A1	S1
Cg	4.95% ± 1.05*	2.38% ± 1.16*#	1.75% ± 0.67*	7.22% ± 4.18*	3.43% ± 2.76*#	3.37% ± 1.64*#
TFC	22.36% ± 3.99	27.32% ± 2.45	29.19% ± 4.62	24.08% ± 3.12	29.41% ± 1.06	29.13% ± 3.80
TFC-Iso	15.60% ± 2.31	39.75% ± 4.86	22.46% ± 8.22	13.55% ± 3.18	30.66% ± 5.56	31.31% ± 1.00
Iso-TFC	0.79% ± 0.19*#	0.97% ± 0.09*#	0.53% ± 0.19*	1.58% ± 0.09*	0.84% ± 0.28*#	0.90% ± 0.55*#

*Data for each behavioral group is presented as the percentage of neurons expressing intranuclear foci of transcription ± SEM. Data for each immediate-early gene is shown by brain region: dorsal hippocampal CA1, primary auditory cortex A1, and primary somatosensory cortex S1. Group sizes: *Cg* = 2, *TFC* = 3, *TFC-Iso* = 3, *Iso-TFC* = 3. ^∗^Statistically significant vs. TFC and ^#^statistically significant vs. TFC-Iso using Tukey-Kramer *post hoc* tests.*

Consistent with previous findings (Vazdarjanova et al., 2002b, 2006; Carpenter-Hyland et al., 2010), the percentage of neurons expressing *Arc* at baseline (Cg group) was low in all examined regions (CA1 = 4.95% ± 1.05, A1 = 2.38% ± 1.64, S1 = 1.75% ± 0.67, **Figures 5A–C**). **Figure 5A** shows that *Arc* expression in dorsal hippocampal CA1 varied dramatically across groups [*F*(3,7) = 14.98, *p* < 0.01]. The TFC group showed significantly higher *Arc* expression than Cg or Iso-TFC groups, but no significant difference compared with the TFC-Iso group. A similar pattern of *Arc* expression was observed in primary auditory cortex, A1 [*F*(3,7) = 39.19, *p* < 0.0001, **Figure 5B**] and primary somatosensory cortex, S1 [*F*(3,7) = 7.63, *p* = 0.013, **Figure 5C**]. Thus, general anesthesia with isoflurane prior to and during training prevented *Arc* expression in all of the examined brain regions, while, significantly, post-training general anesthesia did not stop transcription that was already initiated.

To test whether the observed effects of isoflurane are specific to *Arc* expression, we investigated the expression of another activity-induced IEG, *Zif268*, in the same tissue. ZIF268 protein is a transcription factor known to show an activity-induced pattern of mRNA expression and is also linked to plasticity (Tischmeyer and Grimm, 1999; Hall et al., 2001). In all brain regions there were significant group differences in *Zif268* expression [CA1: *F*(3,7) = 12.36, *p* < 0.01; A1: *F*(3,7) = 24.41, *p* < 0.001; and S1: *F*(3,7) = 51.61, *p* < 0.001]. As with *Arc*, in all three regions we observed low levels of *Zif268* transcription in the Cg group (CA1 = 7.22% ± 4.18, A1 = 3.42% ± 3.90, S1 = 2.31% ± 1.64). Also like *Arc*, the percentage of *Zif268* positive cells was elevated in the TFC group compared to the caged and the Iso-TFC groups, but not compared to the TFC-Iso group (**Figures 5D–F**). General anesthesia with isoflurane affected *Zif268* and *Arc* expression similarly: it prevented initiation of learning-related IEG transcription while not interfering with transcription that was already initiated by fear conditioning.

## Discussion

This study was designed to examine the effect of general anesthesia with isoflurane on learning-induced IEG transcription by assessing the size of neuronal ensembles involved in learning a TFC task. We made two important observations: first, we saw a lack of significant observable effects of isoflurane on either plasticity-related IEG transcription (**Figure 5**) or learning (**Figures 2B,C**) in animals placed under general anesthesia immediately after training. Isoflurane anesthesia did not cause retrograde amnesia for the TFC task in the TFC-Iso group, nor did it stop the transcription of ongoing learning-induced IEG expression. The second finding was that animals who received TFC training while under general anesthesia (Iso-TFC group) exhibited anterograde amnesia (**Figures 2B,C**), a finding consistent with previous studies (Dutton et al., 2002). More importantly, however, these animals showed a lack of learning-induced IEG expression (**Figure 5**). Together, these two observations make an important contribution to understanding the actions of isoflurane on learning and memory at the cellular level. Our findings show that isoflurane does not interfere with ongoing IEG transcription initiated prior to anesthetic administration and, under these conditions, does not cause retrograde amnesia for memory corresponding to this gene expression. In contrast, our results suggest that surgical levels of isoflurane anesthesia prevent the initiation of plasticity-related IEG transcription and subsequently cause anterograde amnesia.

### Isoflurane does Not Stop Ongoing IEG Transcription

To determine whether or not general anesthesia with isoflurane interferes directly with ongoing activity-induced IEG transcription, we examined the effects of general anesthesia administered immediately after Tone Fear Conditioning (TFC-Iso group), once learning-induced *Arc* and *Zif268* transcription was already initiated. If isoflurane affected transcription initiated by fear conditioning performed less than 1 min prior to the induction of anesthesia, then we should see a decrease in the number of neurons with *Arc* and *Zif268* intranuclear foci of transcription. Even if transcription was initiated and prematurely terminated in some neurons within the first few minutes after TFC, our application of the catFISH technique would not count these neurons as IEG positive for two reasons: first, *Arc* and *Zif268* mRNA is not detectable until approximately 2 min following initiation of transcription; second, our counting method excludes very small foci (appearing on less than three planes) that can represent prematurely halted transcription. We did not observe a significant difference in either freezing behavior or the size of neuronal ensembles actively transcribing *Arc* and *Zif268*. This suggests that the basic mechanisms of transcription for these IEGs are not directly affected by isoflurane. The finding that isoflurane administered after learning did not alter IEG transcription is of particular importance for animal-based research. This study was designed, in part, to determine whether the accepted method of anesthetic administration prior to decapitation as a means of euthanasia in animal research could interfere with the study of IEGs. Our findings show that a single administration of isoflurane prior to euthanasia does not interfere with the IEG transcription initiated prior to anesthesia and, for this behavioral task, does not interfere with IEG-dependent learning. While we did not see any differences between the TFC and TFC-Iso groups there may still be differences in gene expression beyond the scope of this study. Isoflurane does not interfere with ongoing transcription, but could still have effects on translation and, in the case of Arc, targeting of mRNA to synapses.

### Isoflurane Prevents Activity-Induced Initiation of IEG Transcription

We observed that animals maintained at a surgical level of anesthesia prior to and during the fear conditioning procedure (Iso-TFC group) did not show active transcription of *Arc* and *Zif268* in brain regions involved in TFC (**Figure 5**), nor did they acquire a conditioned fear response to either the tone or the foot-shock context. While the behavioral effects of isoflurane have been previously reported, the findings describing the effects of isoflurane on transcription of these IEGs is novel. Because Arc and Zif268 proteins are required for the formation of long-term memory (Bozon et al., 2003; Plath et al., 2006), and their mRNA is short-lived (Waters et al., 1990; Lyford et al., 1995), if mRNA transcription is not initiated as a result of behavioral training, then Arc and Zif268 proteins are not available to enable memory consolidation and learning will not occur. Isoflurane alters the functional connectivity of the CNS to the PNS via the sensory thalamus by decreasing, but not preventing, neuronal firing (Vahle-Hinz et al., 2007; Ying et al., 2009). As *Arc* and *Zif268* are both activity-induced IEGs, the dramatic reduction in transcription we observed is most likely caused by decreased neuronal activity rather than interference with the mechanisms of transcription, especially because we also observed that the same level of anesthesia did not prevent transcription that was already initiated.

### Isoflurane has Persistent Effects on Gene Expression: Implications for Lasting Effects on Memory

In addition to the immediate effects of isoflurane on transcription of IEGs, it has an indirect effect on the transcription, as well as the translation, of many other genes. Isoflurane administration results in prolonged upregulation and down-regulation of mRNA and protein expression seen across many genes in various organs (Hamaya et al., 2000; Culley et al., 2006; Rampil et al., 2006; Pan et al., 2008; Kalenka et al., 2010). These effects persist for weeks to months beyond its clearance from the body and therefore cannot be caused by the direct effects of isoflurane binding with cell surface receptors or interacting with cell membranes (Franks and Lieb, 1994; Campagna et al., 2003; Ying et al., 2009; Garcia et al., 2010). Epigenetic changes induced by isoflurane account for some of these changes and can also contribute to anesthesia-induced memory impairment by affecting the same pathways as *Arc* and *Zif268* (Levenson et al., 2004; Chwang et al., 2006; Ahn et al., 2008; Zhong et al., 2014). The proper formation and maintenance of memory requires several rounds of gene expression, making both new and old memories vulnerable to the effects of isoflurane.

Our finding that general anesthesia with isoflurane prevents initiation of transcription of both effector IEGs (*Arc*) and transcription factors (*Zif268*) is of particular significance, because it sheds light on how isoflurane, and possibly other anesthetics, may indirectly alter gene expression in the brain over a period of days to months following anesthesia. If an activity-dependent transcription factor such as *Zif268* is effectively prevented from being transcribed for a period of several hours, during surgery for example, then there will likely be less Zif268 protein available to act as a transcription factor at some time point after anesthetic administration is discontinued. This could lead to a “ripple effect” of diminished transcription (and thus a diminished availability of mRNA for translation) of a wide variety of genes which use Zif268 as a transcription factor. Zif268 is also a transcription factor for itself, so it is plausible that transcription of new *Zif268* mRNA will be affected by the previous decrease in activity and transcription during anesthesia. It is conceivable that a single, discrete period of drastically reduced neuronal activity could have persistent effects throughout multiple rounds of transcription and translation. *Arc*, for example, undergoes a second round of transcription during memory consolidation (Ramirez-Amaya et al., 2005; Nakayama et al., 2015); this second round of *Arc* transcription could be impaired by a decrease in available Zif268 protein due to a decrease in *Zif268* mRNA during anesthesia. A period of drastically decreased neuronal activity has the potential to effect any cellular mechanism that is effected at any stage by the availability of activity-induced gene products. This proposed process for explaining delayed effects from anesthesia may deserve further research.

## Conclusion

We found that isoflurane did not stop ongoing transcription of IEG mRNA but it did prevent the initiation of new activity-induced IEG transcription. These findings correlate well with the observed effects on long-term memory: lack of retrograde amnesia with marked anterograde amnesia. Together, they contribute to understanding the molecular mechanisms of the action of isoflurane. Our findings are important for two reasons: first, this suggests that isoflurane affects protein levels of plasticity-related genes, to a large degree, by decreasing mRNA transcription, and second, brief isoflurane administration prior to euthanasia should not interfere with studies of gene expression. The fact that isoflurane does not interfere with gene transcription when administered immediately before euthanasia is reassuring to those of us who routinely use this method when studying IEGs and may encourage researchers who use live decapitation to employ this method for humane euthanasia.

## Author Contributions

KB, RN, and AV each participated in experimental design, conducted experiments, analyzed data, and helped prepare the manuscript.

## Disclaimer

The views expressed in this article are those of the authors and do not necessarily reflect the position or policy of the Department of Veterans Affairs or the United States government.

## Conflict of Interest Statement

The authors declare that the research was conducted in the absence of any commercial or financial relationships that could be construed as a potential conflict of interest.
