# Supplementary material for: Influence of Isoflurane on Immediate-Early Gene Expression
Source: Front Behav Neurosci. 2016 Jan 12;9:363. doi: 10.3389/fnbeh.2015.00363 (PMC4709487; doi:10.3389/fnbeh.2015.00363)
Supplement: Supplementary file 1 [file Table_1.DOCX]

Supplementary Material

Influence of isoflurane on Immediate-Early Gene expression

Kristopher M. Bunting^1, 2^, Rebecca I. Nalloor^1, 2^, Almira Vazdarjanova^1, 2^

^1^Department of Pharmacology and Toxicology, Medical College of Georgia, Augusta University, Augusta, GA

^2^Charlie Norwood VAMC, Augusta, GA

*** Correspondence:** Almira Vazdarjanova, Department of Pharmacology and Toxicology, Augusta University, CB3526, 1120 15^th^ Street, Augusta GA 30912

avazdarjanova@gru.edu

**Supplementary Table 1**

**Total Neurons Counted**

Data for each behavioral group is presented as the total number of neurons counted using unbiased stereological methods. Data for each immediate-early gene is shown by brain region: Dorsal Hippocampal CA1, Primary Auditory Cortex A1, and Primary Somatosensory Cortex S1.

|  |  | ***Arc*** | | |  | ***Zif268*** | | |
| --- | --- | --- | --- | --- | --- | --- | --- | --- |
| **Group** |  | **CA1** | **A1** | **S1** |  | **CA1** | **A1** | **S1** |
| Cg |  | 987 | 1254 | 971 |  | 1064 | 1147 | 938 |
| TFC |  | 1429 | 1961 | 2179 |  | 1436 | 1835 | 2176 |
| TFC-Iso |  | 1533 | 1632 | 1998 |  | 1554 | 1632 | 2094 |
| Iso-TFC |  | 1258 | 1634 | 1664 |  | 1541 | 1835 | 1979 |
